# Characterization of miR-206 Promoter and Its Association with Birthweight in Chicken

**DOI:** 10.3390/ijms17040559

**Published:** 2016-04-14

**Authors:** Xinzheng Jia, Huiran Lin, Bahareldin Ali Abdalla, Qinghua Nie

**Affiliations:** 1 Department of Animal Genetics, Breeding and Reproduction, College of Animal Science, South China Agricultural University, Guangzhou 510642, China; xinzhengjia@126.com (X.J.); lotuslhr@163.com (H.L.); abdalla406@163.com (B.A.A.); 2 Guangdong Provincial Key Lab of Agro-Animal Genomics and Molecular Breeding, and Key Laboratory of Chicken Genetics, Breeding and Reproduction, Ministry of Agriculture, Guangzhou 510642, China

**Keywords:** miR-206, MyoD, mutation, expression, birthweight

## Abstract

miRNAs have been widely investigated in terms of cell proliferation and differentiation. However, little is known about their effects on bird growth. Here we characterized the promoter of miR-206 in chicken and found that the preferable promoter was located in 1200 bp upstream of pri-miR-206. In this region, many key transcription factors, including MyoD, c-Myb, CEBPα/β, AP-4, RAP1, Brn2, GATA-1/2/3, E47, Sn, upstream stimulatory factor (USF) and CdxA, were predicted to bind and interact with miR-206 promoter. Overexpression of MyoD sharply increased miR-206 expression in both fibroblast and myoblast cells, and also the regulation in the myoblast cells was much stronger, indicating that miR-206 was regulated by MyoD combined with other muscle specific transcriptional factors. Aiming to further investigate the relationship between miR-206 mutation and transcriptional expression, total of 23 SNPs were identified in the two distinct bird lines by sequencing. Interestingly, the motif bound by MyoD was individually destroyed by G-to-C mutation located at 419 bp upstream of miR-206 precursor. Co-transfecting MyoD and miR-206 promoter in DF-1 cells, the luciferase activity of promoter containing homozygous GG types was significantly higher than CC ones (*p* < 0.05). Thus, this mutation caused low expression of miR-206. Consistently, eight variants including G-419C mutation exhibited a great effect on birthweight through maker-trait association analysis in F2 population (*p* < 0.05). Additionally, the regulation of miR-206 on embryo muscle mass mainly by increasing MyoG and muscle creatine kinase (MCK) expression (*p* < 0.05) with little change in MyoD, TMEM8C and myosin heavy chain (MHC). In conclusion, our findings provide a novel mutation destroying the promoter activity of miR-206 in birds and shed new light to understand the regulation mechanism of miR-206 on the embryonic muscle growth.

## 1. Introduction

MicroRNAs (miRNA) are defined as functional noncoding small RNAs, which mainly regulate target genes at transcriptional or post-transcriptional level by specially recognizing and binding to the untranslated regions or coding regions [1,2,3,4]. The classic mode of miRNA action is that miRNA binds to their targets and forms a stable RNA folding structure with much perfect complementarity in the seed sequences (2–8 nt at the 5′ end) of miRNA, and then induces mRNA degradation *in vivo* [5]. In other cases, the miRNA merely inhibits the protein level with little mRNA change. It has been well documented that miRNA have an important function in most biological process including cell proliferation, cell differentiation, cell death, tumorigenesis and so on [6].

Similar to mRNA, most miRNAs have their independent transcription units, which could be initially transcribed by RNA polymerase II to generate the primary transcripts containing cap structures as well as poly(A) tails like protein coding genes [7], and then spliced into mature miRNAs. While, those miRNAs located in genic regions (exon or intron) were was recognized to be transcribed in parallel with their host transcripts [5]. Many investigations have reported that malfunction or abnormal expression of a key miRNA might cause the appearance of disease or special phenotype. miR-9 was recognized as the exhibit hallmarks of spinal muscular atrophy [8]. Using next-generation sequencing to detect the miRNAs expression profile, 12 changed miRNAs were proved as biomarkers in the diagnosis of Alzheimer disease [9]. In cancers, a five-microRNA signature was characterized as an independent predictor of the cancer relapse and survival of non-small cell lung cancer (NSCLC) patients. The patients with high-risk-score in their microRNA signatures had poor overall and disease-free survivals compared to the low-risk-score patients [10]. However, until now little is known about the regulation mechanism of crucial miRNA genes on growth.

So far, several muscle-specific miRNAs were identified to play a crucial role on the muscle cell proliferation, differentiation, contractility and stress responsiveness [11]. Among these muscle-specific miRNAs, the most widely studied miRNAs were members of the miR-1/206 and miR-133a/133b families which highly enriched in both human and mouse heart and skeletal muscle [12]. Many studies also demonstrated that miR-1/206 and miR-133a/133b families were necessary for proper skeletal and cardiac muscle development, and had a profound function on multiple myopathies, such as hypertrophy, dystrophy, and conduction defects [13]. Although miR-1 and miR-206 have the completely conservable seed sequences among all vertebrates, they exhibited different functions during the muscle formation. miR-206 is proved to have much higher expression level than that of miR-1 during development and perinatal period, but in adult muscle it was much lower than that of miR-1 [14,15,16]. During embryonic development in mice, miR-206 was detected at very low level as early as 9.5 days and from about 11.5–12.5 days, its expression sharply increased to high levels until it was born [16]. The expression of miR-206 appears to reach the peak level at 3 days postnatal and then begins to decline [14]. Meanwhile, miR-206 was proved to be significantly increased during the muscle differentiation process *in vivo* or vitro investigations [17]. It indicated miR-206 potentially played various roles in embryo development and postnatal growth.

In this study, we focus on characterizing the promoter of miR-206 in chicken to better understand the regulatory mechanism of miR-206 on the embryonic skeletal muscle growth. We found that the promoter activity of miR-206 mainly lies in about 1200 bp upstream regions of pre-miR-206 and sharply increased in myoblast cell compared to non-muscle fibroblast cell. Interestingly, through analysis of the functional promoter segments, several crucial transcriptional regulation factors were predicted to directly control the genesis of primary miR-206, including the muscle specific transcriptional activator MyoD. *In vitro*, over-expression of MyoD could significantly up-regulate the promoter activity. On the other hand, a large F2 population was designed to analyze the genetic effect of variants in the promoter of miR-206. The results demonstrated that one mutation just destroyed the motif element bound by MyoD resulting in decreased transcriptional activity of miR-206, and also these mutations were significantly associated to birthweight, indicating a potential link between prenatal growth and mutations in the cis-regulate elements of miR-206.

## 2. Results

### 2.1. The Identification of the Promoter Region of gga-miR-206

miR-206 is considered as a skeletal muscle specific expressed miRNA, which plays a crucial role on the skeletal muscle differentiation and myogenesis. The expression of mature miRNA was merely dependent on two steps including pri-miRNA transcriptional process and pre-miRNA splicing matters. Therefore, it is necessary to dissect the promoter of pre-miR-206 to understand the effective transcriptional regulation elements. Through predicting by TFSEARCH 1.3 system (Parallel Application TRC Laboratory, RWCP, Gokasho, Japan) [18], several muscle special transcriptional factors (threshold > 85.0 point) were found in the 1500 bp upstream region, especially concentred in 1000 bp upstream of pre-miR-206. Two elements (−803 and −418 bp) were predicted to be recognized by MyoD, an essential factor for skeletal myogenesis and development (Figure S1). In addition, c-Myb, CEBPα/β, AP-4, RAP1, Brn2, GATA-1/2/3, E47, Sn, upstream stimulatory factor (USF) and CdxA were identified to bind to different regions of the miR-206 promoter, most of which were reported to be related to muscle growth and differentiation.

**Figure 1:**
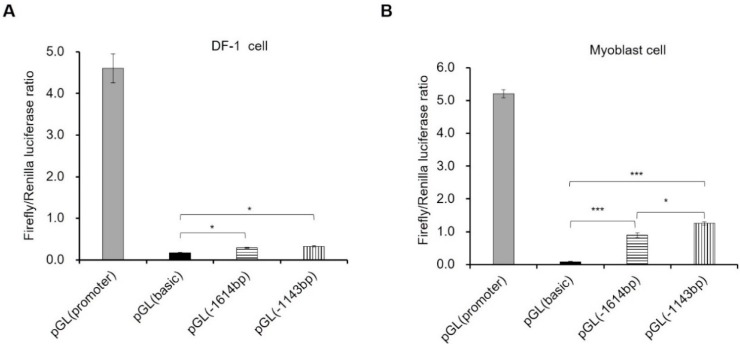
Characterization of promoter activity of gga-miR-206 in DF-1 and myoblast cell. (**A**) Detection of miR-206 promoter activity in DF-1 cell lines; (**B**) Detection of miR-206 promoter activity in myoblast cells. Myoblast cell was isolated from skeletal muscle of 11-day-old embryos. The ratio of firefly and renilla luciferase was used for detecting the promoter activity through co-transfecting pGL3.0 and pGL-TK (as normalize control). In this panel, data are presented as mean ± standard error (SE). * *p *< 0.05 and *** *p *< 0.001 were estimated by Student’s *t*-test (*n *= 3).

In order to experimentally confirm the core promoters of miR-206, two distinct length of upstream regions (1614 and 1143 bp) of pre-miR-206 were cloned into pGL3.0-basic vector to detect the promoter activity. After co-transfecting with pGL-TK in DF-1 cell, both of pGL(−1614 bp) and pGL(−1143 bp) presented less than 10% activity of stronger promoters pGL(promoter) and pGL(−1614 bp) showed a slightly increasing promoter activity compared to pGL(basic) negative control (*p *< 0.05) (Figure 1A). No significant difference was found between pGL(−1614 bp) and pGL(−1143 bp) (*p *> 0.05). However, in myoblast cells, both pGL(−1614 bp) and pGL(−1143 bp) presented more than 24% activity of stronger promoter pGL(promoter), and displayed a significantly increasing promoter activity compared to negative control (*p *< 0.001 and *p *< 0.05, respectively) (Figure 1B). These data proved that the region at about 2000 bp upstream had a certain promoter activity, and the activity in myoblast cell was much stronger than that in DF-1 cell, which suggested that the transcription of pri-miR-206 might be regulated by muscle specific transcriptional factor. Moreover, this region might exist some no muscle specific regulated elements since a slight promoter activity were detected in DF-1 cell. In myoblast cell, the activity of pGL(−1143 bp) were significant higher than that of pGL(−1614 bp), which implied that there were some muscle special silence elements located in the region from −1143 to −1614 bp, and the core promoter was located in about 1100 bp upstream of miR-206.

### 2.2. MyoD up-Regulates the Transcriptional Activity of gga-miR-206 in Vitro

**Figure 2:**
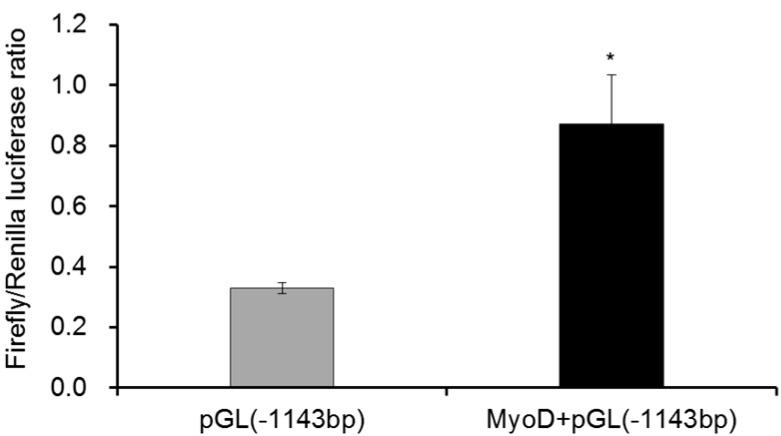
Overexpression of MyoD significantly increased the promoter activity in DF-1 cell. The whole promoter (about 1200 bp) of miR-206 were recombined into pGL3.0-promoter plasmid, and con-transfecting pGL-TK and pGL-promoter with pcDNA-MyoD or control pcDNA vectors were performed to detect the promoter activity *in vitro*. In this panel, data are presented as mean ± standard error (SE). * *p *< 0.05 was estimated by Student’s *t*-test (*n* = 3).

In order to confirm the regulatory relationship between MyoD and gga-miR-206 transcription, over-expression vector of pcDNA3.1-MyoD was constructed to analysis the promoter activity in DF-1 cells. After overexpression of MyoD in DF-1 cell for 36 h, the firefly luciferase (normalized to renilla luciferase activity) was significantly increased (*p *< 0.05) by nearly three folds (Figure 2). It suggested that MyoD could directly enhance miR-206 transcriptional process by binding to the promoter elements as predicted by this study.

### 2.3. MyoD Induces Muscle-Specific miR-206 Expression in Fibroblast and Myoblast Cells

**Figure 3:**
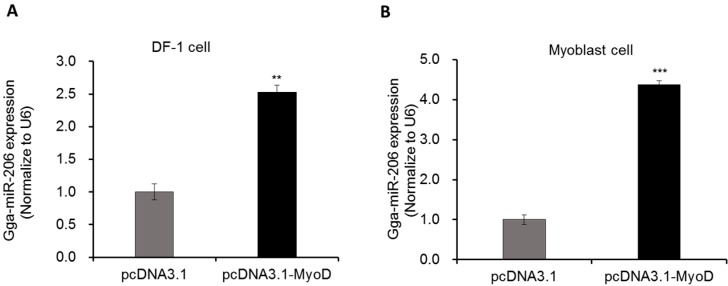
The effects of MyoD on miR-206 expression. (**A**) Overexpression of MyoD significantly increased miR-206 expression in fibroblast; (**B**) Overexpression of MyoD significantly increased miR-206 expression in myoblast cells. After transfecting pcDNA-MyoD or control pcDNA3.1 vector for 36 h, miR-206 relative expression were detected by qPCR. U6 was used for referenced gene. In this panel, data are presented as mean ± standard error (SE). ** *p *< 0.01 and *** *p *< 0.001 were estimated by Student’s *t*-test (*n* = 3).

miR-206, as a muscle-specific expressed miRNA, is largely acknowledged as a positive regulator of skeletal muscle differentiation and growth. Until now, no investigation was reported that miR-206 expressed in other non-muscle tissues. Here, we tried to over-express MyoD in chicken non-muscle DF-1 fibroblast cells to explore the miR-206 transcription mechanism. The results showed that mature miR-206 was significantly up-regulated (*p *< 0.01) in fibroblast cell after transfecting pcDNA-MyoD for 36 h (Figure 3A). In primary chicken myoblast cell, the expression of miR-206 was great significantly increased (*p *< 0.001) after transfecting pcDNA-MyoD for 36 h, in which the fold change was much higher than in fibroblast cells (Figure 3B).

### 2.4. Polymorphisms in the Promoter Region of gga-miR-206

Gga-miR-206 was reported to differently express in embryonic skeletal muscle tissues between meat-type broilers and egg-type layer chickens [19]. For a comprehensive understanding of the expression pattern in different breeds, it is necessary to identify the polymorphisms and predict the transcriptional motifs in the promoter region of pre-miR-206. Here we detected the mutation in its promoter region between fast- and slow-growing breeds (WRR & XH) through directly sequencing. The results showed that a total of 23 SNPs were identified in this region, with one SNP per 100 bp (Table 1). Interestingly, 13 transcriptional factors were affected in various degrees by the change of mutated allele. G-419C and G-804T have destroyed the regulatory elements binding to MyoD. A-908G, C-713G and A-145G were recognized by GATA family factors. C-964G and C-607T were specially binding to c-Myb transcriptional factors. It implied that these mutations might change the expression pattern through these transcriptional factors.

### 2.5. The Mutation G-419C Affects the miR-206’s Promoter Activity

**Figure 4:**
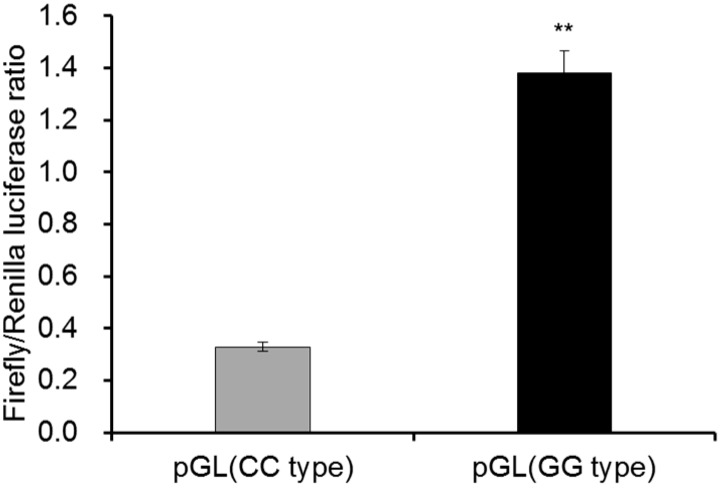
The promoter activities of miR-206 were different between the wild and mutation types. The promoter containing wild (GG) or mutation (CC) phenotype at 419 upstream of precursor of miR-206 were recombined into the pGL3.0-promoter systems to test the promoter activities. pGL-TK was con-transfected as the normalization. In this panel, data are presented as mean ± standard error (SE). ** *p *< 0.01 was estimated by Student’s *t*-test (*n* = 3).

Through comparing the miR-206 promoter region between different chicken breeds, several variations were identified and predicted to affect the regulation process by transcriptional factors, including MyoD. Here, we constructed the wild promoter (GG genotype for G-419C) cloned from domestic XH chicken, and the mutation promoter (CC genotype for G-419C). After transfected into DF-1 cell for 36 h, the promoter activity of mutation type was found to be significantly down-regulated 4-fold (*p *< 0.01) compared to the wild type (Figure 4). According to the binding relationship between MyoD and miR-206 promoter, this SNP (G-419C) from G to C might alter the promoter activity and destroy its binding ability to MyoD. Therefore, the GC mutation in the 419 bp upsteam of miR-206 was able to cause altered expression abundance of primary miR-206 through changing the promoter activity, finally resulting in ectopic expression of funtional miR-206.

### 2.6. Genetic Effects of Variants in the Promoter Region of miR-206 on the Birth Weight

Since the mutations in the promoter of miR-206 were able to change miR-206 expression, it was reasonable to hypothesize that these mutations would have an effect on growth performance, such as birth weight. In order to confirm this, all variants in the promoter of miR-206 were further genotyped in a F2 family resource population to evaluate the genetic effects on chicken growth. Totally, we found that 11 SNPs had a valuable polymorphism distribution. Among them, 8 SNPs, including G-615A, G-515T, A-470T, G-419C, T-356C, A-145G, G-140A, and G-114A, were significantly or greatly significantly associated (*p *< 0.05 or *p *< 0.01) with the chicken birth weight (Table 2). Interestingly, both of G-419C and A-145G, which impacted the binding ability of MyoD and GATA transcriptional factors, respectively, were significantly associated with the birth weight (*p *< 0.05). Our findings revealed that mutations in the promoter of miR-206 might influence the embryo development, resulting in significant differential birth weight.

### 2.7. miR-206 Partly Induced Myogenesis by Myog and MCK

**Figure 5:**
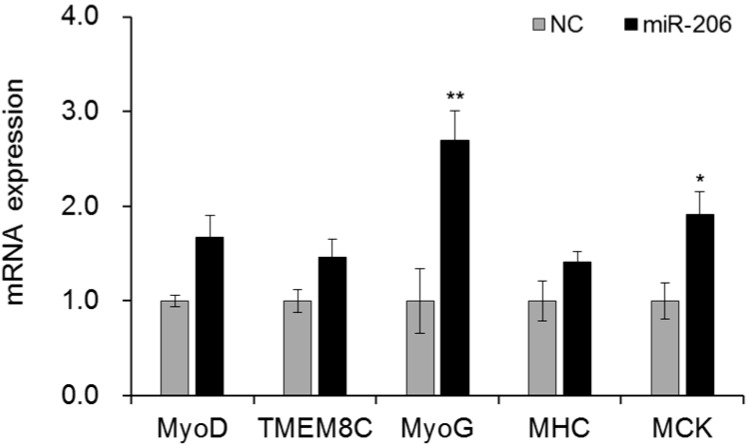
The expression profiles of muscle differentiation marker genes were regulated by miR-206 in the chicken embryo myoblast cells. Mimic miR-206 and negative control (NC; random sequence molecules) were transfected into the cells for 36 h to test the mRNA expression level of the maker genes. β-actin was used as the referenced gene. In this panel, data are presented as mean ± standard error (SE). * *p *< 0.05 and ** *p *< 0.01 were estimated by Student’s *t*-test (*n* = 3).

miR-206, as a muscle specific miRNA, integrates multiple components in differentiation pathways to control the muscle growth. Here, we explored whether miR-206 could impact on the mRNA expression of the muscle differentiation marker genes, such as MyoD, TMEM8C, MyoG, myosin heavy chain (MHC) and muscle creatine kinase (MCK). After over-expression of miR-206 in myoblast cell for 36 h, MyoG and MCK were significantly up-regulated (*p *< 0.05 or *p *< 0.01) by 2.5- and 1.9-folds, respectively (Figure 5). However, the other three maker genes showed no big change. These data suggested that during the early stage (36 h) of myoblast growth, the role of miR-206 mainly through directly or indirectly regulating MyoG and MCK rather than MyoD, TMEM8C and MHC at the transcription level.

## 3. Discussion

Muscle specific miRNAs, including miR-1/206 and miR-133b, are well characterized to play crucial roles in muscle development in human and mouse [20,21,22,23,24]. A previous study demonstrated that miR-206 was involved in the muscling phenotype in growth-selected chicken lines [25]. However, little is known about miR-206 promoter and its effects on miRNA expression at the transcriptional level in birds. In the present study, miR-206 was predicted to be regulated by the muscle special transcriptional factors. We showed that miR-206 had independent promoter and the prefer promoter activity was in the 1200 bp upstream region of pre-miR-206. Among those identified transcription factors binding to regulatory elements, such as MyoD, c-Myb, CEBPα/β, AP-4, RAP1, Brn2, GATA-1/2/3, E47, Sn, USF and CdxA, most were reported to be relative to muscle growth and differentiation [17,26,27,28]. MyoD is capable of converting fibroblast cell into terminally differentiated skeletal muscle [29]. In addition, it was proved that MyoD could interact with RUNX1 and ZNF238 to control pri-miR-206’s transcription [30]. Another key regulator, GATA1, could control the activity of MEF2A through a dominant-negative mechanism and played an essential role in muscle differentiation during embryogenesis and adult regeneration [31,32]. Interestingly, in this study, we identified two MyoD binding sites and one GATA1 binding site in the miR-206 promoter. In addition, the genetic variations G-419C (MyoD) and A-145G (GATA1) occurred at these binding sites. Through genotype and phenotype association analysis, these two mutations were demonstrated to greatly affect the chicken birth weight, indicating that these SNPs might be related to the embryogenesis.

To characterize the promoter activity of miR-206 further, we used pGL3-promoter luciferase report system to detect the promoter ability. The results showed that the 1200 bp upstream region of pri-miR-206 had greater activity, comparing to 1600 bp upstream region. It indicated that the region from 1200 to 1600 bp might negatively regulate the miR-206 promoter activity. In this study, we found C-Myb and CEBP family were binding to this region, and both of them were reported to be related to muscle growth [33,34,35]. Meanwhile, we also detected that the promoter ability altered during the mutation from G to C at 419 bp upstream of pri-miR-206. The promoter activity was down-regulated in CC type, which was in consistent with the bioinformatic prediction that the mutation from G to C destroyed the MyoD binding sites, and finally affected the promoter ability. Considering to the genetic effect on birth weight, here we identified an effective genetic marker for domestic chicken breeding against growth.

MyoD was reported to induce the fibroblast cell to myoblasts [36]. In this study, overexpression of MyoD in fibroblast cell could significantly induced miR-206 expression. Meanwhile, miR-206 was low endogenously expressed in embryo fibroblast cells. In myoblast cell, the miR-206 expression was obviously up-regulated after transfecting MyoD for 36 h. It suggested that in the muscle cells, the miR-206 was also promoted by other special transcriptional factors except for MyoD. Similar to investigation in the neural tube of chicken embryos, MyoD and MRFs were able to directly activate miR-206 expression, while in the MyoD deleted chicken embryos, they also could detect the miR-206 expression [37]. These results demonstrated that MyoD is not necessary for the miR-206 genesis. Probably, MyoD, as a global muscle transcriptional factor, partly impacted the miR-206 promoter ability through direct and indirect regulation. This point was also proved by the results that the longer promoter region of miR-206 including MyoD binding sites showed lower promoter ability. All these results indicated that the transcription process of primary miR-206 was complex and might involve many regulated factors.

In previous studies, miR-206 greatly promoted the muscle development by multiple pathways. We over-expressed miR-206 in the chicken myoblast cell to detect the change of the maker genes during myogenesis. miR-206 greatly increased MyoG and MCK expression, and MyoD, TMEM8C and MHC exhibited little change. Generally, MyoD, TMEM8C and MyoG were the early differentiation markers expressed in proliferating muscle cells, while the MHC and MCK were the lately differentiation marker genes. miR-206 could induce both MyoG and MCK up-regulation, which suggested that miR-206 might independently promote muscle differentiation. Other maker genes showed no change demonstrated that miR-206 might function through MyoG and MCK pathway to induce the differentiation process.

## 4. Experimental Section

### 4.1. Ethics Statement

All animal experiments were conducted in accordance with regulations of the Administration of Laboratory Animals of Guangdong Province. The Animal experiments were approved by the Animal Care and use Committee at the South China Agricultural University (Guangzhou, China) with approval number SCAU#0011, 3 August 2010.

### 4.2. Animals

An F2 resource population derived from reciprocal crosses between WRR and XH chickens were employed in the present study. WRR chicken is a fast-growing broiler breed and XH chicken is a slow-growing Chinese indigenous breed. Nine females and seven males from XH, and eight females and nine males from WRR were selected for mating. The reciprocal mating of the XH (♂) × WRR (♀) and WRR (♂) × XH (♀) were selected on the basis of satisfactory egg and semen yields to produce the F1 generation. Each male was paired with a female from the other line, except one male from XH, which paired with two females from WRR. At 30 weeks of age, 17 F1 males and 17 F1 females were selected to produce the F2 generation, resulting in a total of 489 birds in 17 full-sib families from six hatches at two-week intervals [38]. All F2 birds were weighted after birth, and recorded data were used for the association analysis between variations and embryo growth traits.

### 4.3. Cell Culture

DF-1 cell line of chicken embryo fibroblast was obtained from the Cell Bank of Committee on Type Culture Collection of the Chinese Academy of Sciences, which was cultured in DMEM (Invitrogen, Carlsbad, CA, USA), with 10% fetal bovine serum (FBS) (Gibico, Grand Island, NY, USA) and 0.2% penicillin/streptomycin (Invitrogen, Carlsbad, CA, USA) in humidified air at 37 °C with 5% CO_2_.

Primary myoblast cells were isolated from the muscle tissue of 11-day chicken embryos. Firstly, the muscle tissues were dissected away from the skin and bone with sterile forceps, and then minced to a slurry with razor blades in the culture dish. During the whole process, the muscle tissues were maintained in the complete DMEM culture including 20% foetal bovine serum, 1% nonessential amino acids and 0.2% penicillin/streptomycin. Then, the suspension was shaken by repetitive vortexing for three times and filtered to remove the large debris. The single cells were collected by centrifugation at 500× *g*. Thirdly, serial plating protocol was used to enrich myoblasts and eliminate fibroblasts. The collected cell suspension was cultured in the complete DMEM culture including 20% foetal bovine serum, 1% nonessential amino acids and 0.2% penicillin/streptomycin. After 40 min, the cell suspension was transferred into a new plate and repeated for three times. Finally, the primary myoblast cells were cultured in the complete DMEM culture including 20% foetal bovine serum, 1% nonessential amino acids, 0.2% penicillin/streptomycin and humidified air at 37 °C with 5% CO_2_.

### 4.4. RNA Extraction and RT-PCR

Total RNA was extracted from collected cells using TRizol (Invitrogen, Carlsbad, CA, USA) according to the manufacturer’s instructions. The extracted RNAquantity was checked by 2% agarose gel electrophoresis and the concentration was determined by measuring the optical density in a Nanodrop 2000 spectrophotometer at 260/280 nm ratio. Reverse transcription (RT) was performed at 37 °C for 30 min in a total volume of 20 μL consisting of 2 μg total RNA and reaction solution provided in the PrimeScript™ RT-PCR Kit (Takara, Otsu, Japan). miRNA first-strand cDNA was synthesized from total RNA (1 μg) using a miScript Reverse Transcription kit (Qiagen, Hilden, Germany) following the manufacturer’s protocols.

### 4.5. Real Time RT-PCR

The mRNA and miRNA expression analysis were carried out on CFX96 (Bio-Rad, Hercules, CA, USA), in which the β-actin and U6 were used as internal control, respectively. The mRNA expression level was determined with the use of real time PCR Master Mix (SYBR Green) Kit (Takara, Otsu, Japan), and miRNA was performed with the miScript SYBR Green PCR kits (Qiagen, Hilden, Germany) including the miScript Universal reverse primers; following the manufacturer’s protocols. The 20 μL reaction mixtures were incubated at 95 °C for 15 min, followed by 40 cycles at 94 °C for 15 s, 58–60 °C for 30 s and 72 °C for 10 s. All primers are presented in Table 3. The relative mRNA level of target gene was calculated by the comparative equation 2^−∆∆*C*t^ (∆∆C_t_ = ∆*C*_t target gene_ − ∆*C*_t reference gene_).

### 4.6. Over-Expressing miR-206 and MyoD in Vitro

In order to gain highly effective overexpression of miR-206, three concentration gradients (20, 40 and 80 nM) of mimic miRNAs (RiboBio, Guangzhou, China) were transfected into DF-1 and myoblast cells for 36 h with 2 µL Lipofectamine 3000 (Invitrogen, Carlsbad, CA, USA). The results showed that 40 nM mimic miR-206 caused better overexpression with more than 500-fold and the 12-fold increasing in DF-1 and myoblast cells, respectively (Figure S2). Therefore, the 40 nM mimic miR-206 was transfected into DF-1 and myoblast cells with 2 µL Lipofectamine 3000. All cells were collected after 36 h to detect the mRNA and miRNA expression.

The coding sequences of *MyoD* were cloned into pcDNA3.1 vector by a pair of primers (Table 3). Then, 0.5 µg pcDNA-MyoD was co-transfected by 1.5 µL Lipofectamine 3000 with 0.5 µg pGL(-1143 bp) and 0.05 µg pGL-TK per well for 24-well plate into DF-1 cell to detect its effect on the miR-206 promoter activity. Next, 3 µg pcDNA-MyoD per well of 12-well plate was transfected by 3 µL Lipofectamine 3000 into primary myoblast and DF-1 cells for 36 h to detect the miR-206 expression.

### 4.7. SNP Identification

Genomic DNAs from 5 random F2 samples of XH & WRR resource population were used as templates. miR206GT primers were used to amplify different regions including miR-206 up-flanking and down-flanking sequences. PCR was performed in 15 µL mixtures containing 50 ng of chicken genomic DNA, 1× PCR buffer, 10 pmol of primers, 100 µM of each dNTP, 1.5 mM MgCl_2_ and 1.0 U Taq DNA polymerase (Takara, Otsu, Japan). PCR was run in three steps methods: 3 min at 94 °C, followed by 34 cycles of 30 s at 94 °C, 30 s at annealing temperature 60 °C, 1 min at 72 °C, and a final extension of 5 min at 72 °C. The PCR products were subjected to directly sequencing by a commercial service (Sangon biotech, Shanghai, China) for SNP identification and genotyping.

### 4.8. Luciferase Reporter Assay for miR-206 Promoter Activity

PGL3.0 luciferase reporter system (Promega, Madison, WI, USA) was used to detect the miR-206 promoter activity. Two fragments with different lengths of upstream sequences were cloned into pGL3.0 vectors. After co-transfected 0.5 µg recombinant vectors and 0.05 µg pGL-TK vector into myoblast and DF-1 cells for 36 h by Lipofectamine 3000, luciferase activity was measured using Dual-Luciferase Reporter Assay System (E1910) as described by the manufacturer’s instructions (Promega, Madison, WI, USA). In each case, renilla luciferase activity was served as a normalization control. For miR-206 over-expression, 40 µM mimic miR-206 was transfected into DF-1 and myoblast cells. All cells were collected after 36 h to detect mRNA and miRNA expression.

### 4.9. Association Analysis and Statistical Analysis

Association analysis was performed using the GLM (General Linear Models Procedures) of Statistical Analysis Software 8.1(SAS 8.1) (SAS Institute Inc., Cary, NC, USA). The genetic effects were analyzed by a general mixed procedure in the SAS package. Student’s *t*-test was used to calculate statistical significance for mRNA level comparisons in varied breeds and treatments in the same group of subjects.

## 5. Conclusions

In conclusion, our results revealed the detailed characters of miR-206 promoter region and identified a causative mutation impacting on the expression level and growth performance in birds. Overexpression of MyoD sharply increased miR-206 expression in both fibroblast and myoblast cells. Aiming to further investigate on the relationship between miR-206 mutation and transcriptional expression, total of 23 SNPs were identified in the two distinct bird lines using sequencing. Interestingly, MyoD binding motifs was individually destroyed by G-to-C mutation located at 419 bp upstream of miR-206 precursor. In DF-1 cells, the luciferase activity of promoter containing homozygous GG types was significantly higher than CC ones (*p *< 0.05), resulting in low expression of miR-206 caused by this mutation. Consistently, 8 variants including G-419C mutation were significantly associated with birthweight in F2 population (*p *< 0.05). Additionally, the regulation of miR-206 on embryo muscle mass mainly by increasing MyoG and MCK expression (*p *< 0.05) with little change in MyoD, TMEM8C and MHC. As a result, our findings provide a novel mutation destroying the promoter activity of miR-206 in the birds and shed a new light to understand the regulation mechanisms of miR-206 on the chicken embryonic muscle growth.

## Figures and Tables

**Table 1:** Variants were identified in the promoter region of miR-206 in broilers.

Maker	Position	Chr Location	Type	Sequences
SNP1	−1020 bp	107215055	AT	AACCAAAATGACTGGGTGTGA
SNP2	−964 bp	107214999	CG	AGTTGCGAAACAGAAAGTTTCT
SNP3	−908 bp	107214943	AG	GGTAGGATGAAGAATCCCATG
SNP4	−804 bp	107214839	GT	TGGGACCTCTGGGTCCATCTG
SNP5	−713 bp	107214748	CG	AGAAGAGATACTCCACTACCT
SNP6	−674 bp	107214709	CA	ATGAAGAAGACACAGGAGATA
SNP7	−615 bp	107214650	GA	TGGTTGCAATGCATCAGGTTA
SNP8	−607 bp	107214642	CT	ATGCATCAGGCTAACTTCCCT
SNP9	−569 bp	107214604	CT	AAGACATTTCCACTTAGGACA
SNP10	−515 bp	107214550	GT	ACTCCAACCGGTTTTTTCCCA
SNP11	−470 bp	107214505	AT	ATCTCCAGCAACCCTGAGTGT
SNP12	−454 bp	107214489	CT	AGTGTAGCTGTCCCATCAAGA
SNP13	−419 bp	107214454	GC	CAAACCAGGTGCTCCAGTAGA
SNP14	−390 bp	107214425	CG	AAGGGAGGGACAGTGGTGCCA
SNP15	−356 bp	107214391	TC	CCAATTGACCTAAGCTTGACC
SNP16	−327 bp	107214362	GT	GGCAAAGAGAGGGACAAGAGG
SNP17	−244 bp	107214279	GA	GAACCAGACCGGGCTCCAGTG
SNP18	−145 bp	107214180	AG	CTTCCTGATCAGGACATTTGT
SNP19	−140 bp	107214175	GA	TGATCGGGACGTTTGTACCAA
SNP20	−120 bp	107214155	CT	ATAATAATAACATTTTGGTGT
SNP21	−114 bp	107214149	GA	ATAATATTTTGGTGTTCCTGC
SNP22	−83 bp	107214118	AG	AGGAGAAAGCAGATCACCAGC
SNP23	−32 bp	107214067	CT	TCTCCAGGAGCGCCCAGAGGT

**Table 2:** The association analysis was performed on miR-206 mutation and birthweight.

SNPs	Position	Mutation	Birthweight (Least Squares Mean, g)			*p* Value
SNP7	−615 bp	G/A	30.1424 (GG/66)	31.7435 (GA/23)	29.4868 (AA/189)	0.0035
SNP10	−515 bp	G/T	29.5055 (GG/183)	30.8324 (GT/34)	30.2292 (TT/72)	0.0355
SNP11	−470 bp	A/T	31.0194 (AA/36)	30.9600 (AT/15)	29.6075 (TT/240)	0.0232
SNP12	−454 bp	C/T	30.1040 (CC/151)	30.5220 (CT/41)	29.2430 (TT/100)	0.1726
SNP13	−419 bp	G/C	30.8111 (CC/36)	29.1286 (CG/27)	29.0522 (GG/249)	0.0229
SNP15	−356 bp	T/C	30.1899 (TT/69)	31.0147 (TC/34)	29.5439 (CC/189)	0.0138
SNP17	−244 bp	G/A	30.5969 (GG/129)	30.555 (AG/40)	28.8797 (AA/123)	0.1202
SNP18	−145 bp	A/G	31.0194 (AA/36)	31.25 (AG/16)	29.6203 (GG/241)	0.0163
SNP19	−140 bp	G/A	31.0194 (AA/36)	31.25 (AG/16)	29.6203 (GG/241)	0.0163
SNP20	−120 bp	C/T	28.9643 (CC/126)	30.5429 (CT/42)	30.6032 (TT/126)	0.2140
SNP21	−114 bp	G/A	31.0194 (AA/36)	31.25 (AG/16)	29.6203 (GG/241)	0.0163

Statistical analysis was performed using GLM by SAS 8.0, and the different phenotype individuals were shown the least squares mean and sample numbers. *p *< 0.05 indicated significant association, and *p *< 0.01 indicated highly significant association.

**Table 3:** All primers were used in this study.

Primer Name	Primer Sequences (5′→3′)	Accession Number	Product Size (bp)	Tm (°C)	Notes
miR206-pro-F1	ggggtaccggcatcaccttgctaccctaaa	NR_031431.1	1592	65	pGL-vector
miR206-pro-R1	ccgctcgagagaggcagcatttctcctcatc				
miR206-pro-F2	ggggtaccgcccatttccttcaacctttcc	NR_031431.1	1121	65	pGL-vector
miR206-pro-R2	ccgctcgagagaggcagcatttctcctcatc				
miR206GT-F	cataaatcgtggaacaacgcataa	NR_031431.1	1198	62	SNP
miR206GT-R	gcagtagctggaagcagaggac				
myoD-F2	ggggtaccactccgacgttcccagtc	NM_204214.2	1213	62	pcDNA-MyoD
myoD-R2	cggaattccaggttccctattctccaaa				
MyoD-F	ggaaggaggaaacctgagtga	NM_204214.2	129	60	qPCR
MyoD-R	ctggacctgcctttatagcac				
MYOG-F	cggaggctgaagaaggtgaa	NM_204184.1	219	60	
MYOG-R	cggtcctctgcctggtcat				
MHC-F	ctcctcacgctttggtaa	NM_001319304	218	60	
MHC-R	tgatagtcgtatgggttggt				
MCK-F	ctgggcttctcggaggtgga	NM_205507.1	109	60	
MCK-R	cgtctatgggctggttctgct				
TMEM8C-F	atcgaccttcatcatgtttgg	NM_001318457.1	161	60	
TMEM8C-R	ttgtctggatacagccctttc				
gga-miR-206	tggaatgtaaggaagtgtgtgg	NR_031431.1	65	58	
U6-qiagen-F	cgatacagagaagattagcatgg cccctgc	EU240275	109	58	
β-Actin-F	ctcccccatgccatcctccgtctg	NM_205518.1	179	60	
β-Actin-R	gctgtggccatctcctgctc				

Notes indicated the position of cloning segments of miR-206 genes. miR-206 and U6 reversed primers were provided in the miScript SYBR Green PCR kits (Qiagen, Hilden, Germany).

## Supplementary Materials

Supplementary materials can be found at http://www.mdpi.com/1422-0067/17/4/559/s1.
