# Supplementary material for: Characterization of miR-206 Promoter and Its Association with Birthweight in Chicken
Source: Int J Mol Sci. 2016 Apr 14;17(4):559. doi: 10.3390/ijms17040559 (PMC4849015; doi:10.3390/ijms17040559)
Supplement: Supplementary file 1 [file ijms-17-00559-s001.pdf]

SupplementaryMaterials: Characterization of miR-206 Promoter and Its Association with Birthweight in Chicken

Xinzheng Jia, Huiran Lin, Bahareldin Ali Abdalla and Qinghua Nie

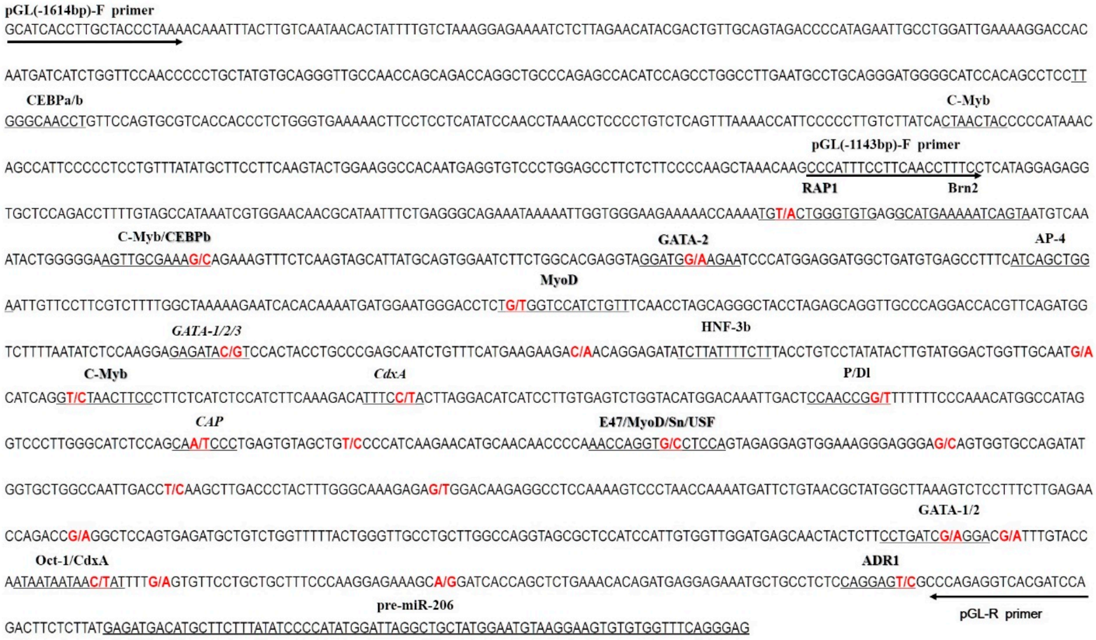

Figure S1. Transcriptional regulatory elements and mutations in the promoter of gga-miR-206. The underline means the regulatory elements recognized by related transcriptional factors, which were predicted by TFSEARCH 1.3 system [18] with the threshold more than 85.0 points in the 1200 bp upstream region. Bases marked in red text mean a point mutation. Arrows indicate three primers used for promoter amplification.

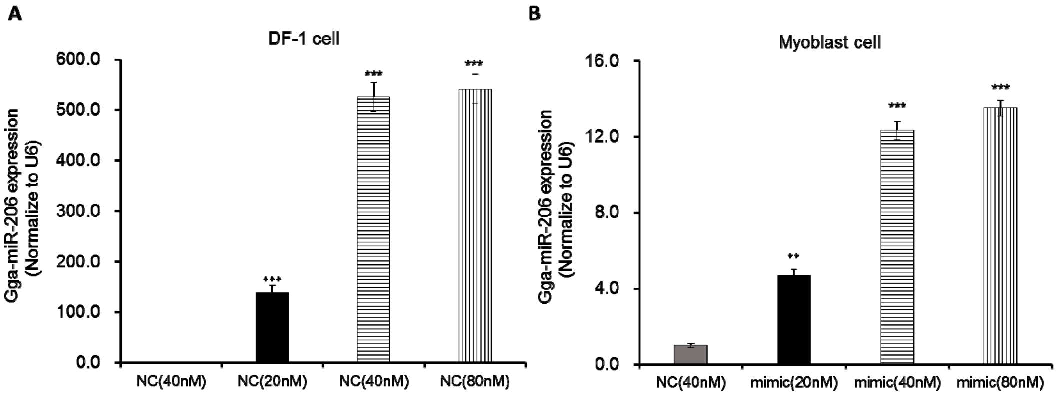

Figure S2. (**A**) The expression changes of miR-206 were induced by various concentration of mimics in DF-1; (**B**) The expression changes of miR-206 were induced by various concentration of mimics in and myoblast cells. Three concentration gradient of mimic miRNAs (20, 40 and 80 nM) were transfected into DF-1 and myoblast cells for 36 h. In this panel, data are presented as mean ± standard error (SE). ** $p < 0.01$ and *** $p < 0.001$ were estimated by Student's t-test ($n = 3$).
